# The Influence of Concurrent Autoimmune Thyroiditis on the Cardiometabolic Consequences of Cabergoline in Postmenopausal Women

**DOI:** 10.3390/metabo15010009

**Published:** 2025-01-01

**Authors:** Robert Krysiak, Marcin Basiak, Witold Szkróbka, Bogusław Okopień

**Affiliations:** Department of Internal Medicine and Clinical Pharmacology, Medical University of Silesia, 40-752 Katowice, Poland; rkrysiak@sum.edu.pl (R.K.); wszkrobka@sum.edu.pl (W.S.); bokopien@sum.edu.pl (B.O.)

**Keywords:** insulin sensitivity, lactotroph, prolactin excess, postmenopause, thyroid autoimmunity

## Abstract

**Background:** Untreated hyperprolactinemia and autoimmune thyroiditis (Hashimoto’s disease) seem to increase cardiometabolic risk. The cardiometabolic effects of cabergoline were less significant in young women with concurrent euthyroid Hashimoto’s illness. This study sought to investigate if the detrimental effects of this condition on cabergoline efficacy are also evident in postmenopausal women. **Methods:** The study comprised 50 postmenopausal women exhibiting increased prolactin levels, with half qualifying for euthyroid Hashimoto’s illness. The subjects with thyroid autoimmunity were matched with those without thyroid disease according to age, body mass index, and prolactin levels. In addition to prolactin, we assessed thyroid-stimulating hormone (TSH), thyroid antibodies, and glucose homeostasis markers: fasting glucose, the homeostatic model assessment 1 of insulin resistance ratio (HOMA1-IR), and glycated hemoglobin (HbA1c). Furthermore, we assessed plasma lipids, plasma uric acid levels, high-sensitivity C-reactive protein (hsCRP), fibrinogen, homocysteine, and the urine albumin-to-creatinine ratio (UACR). The decadal cardiovascular risk was assessed with the Framingham Risk Score (FRS). **Results:** Before therapy, disparities existed among groups in HOMA1-IR, HDL cholesterol, antibody titers, uric acid, hsCRP, fibrinogen, homocysteine, UACR, and FRS. After six months of treatment, cabergoline successfully corrected prolactin levels (both total and monomeric) in women without thyroid disorders. This normalization correlated with decreases in HOMA1-IR, triglycerides, uric acid, hsCRP, fibrinogen, homocysteine, UACR, and FRS, as well as an elevation in HDL cholesterol. In women diagnosed with Hashimoto’s disease, cabergoline’s effects were limited to a reduction in prolactin levels, HOMA1-IR, and UACR, as well as an elevation in HDL cholesterol, with these alterations being less pronounced compared to women without thyroid illness. **Conclusions:** The cardiometabolic benefits of cabergoline were associated with the degree of prolactin concentration reduction. In women diagnosed with Hashimoto’s disease, connections were noted between baseline levels and treatment-induced alterations in hsCRP. These data indicate that concurrent euthyroid autoimmune thyroiditis mitigates the cardiometabolic consequences of cabergoline.

## 1. Introduction

Because of the lack of classic symptoms, such as amenorrhea and galactorrhea, prolactin excess after menopause is rarely diagnosed and often untreated [1]. In numerous studies, hyperprolactinemia was accompanied by impaired insulin sensitivity, excessive fat accumulation, systemic inflammation, endothelial dysfunction, arterial stiffness, and enhanced coagulation [2,3,4,5,6,7,8,9]. These negative outcomes were absent or less pronounced in individuals receiving a dopamine agonist, particularly cabergoline [2,3,5]. Following the discontinuation of cabergoline medication in patients with prolactin-secreting tumors, there was a concomitant rise in both prolactin and fibrinogen levels [10]. This suggests that patients with prolactin excess may be more prone to cardiovascular and metabolic complications, as well as that the development of these complications may be prevented by treatment with dopaminergic agents. However, it should be underlined that studies assessing risk factors included only or predominantly young, symptomatic women, and that unfavorable effects may be, at least partially, associated with hyperprolactinemia-induced reductions in gonadotropin secretion resulting in subsequent hypogonadotropic hypogonadism [1]. However, though physiological gonadal failure after menopause by increasing cardiometabolic risk in healthy postmenopausal women may theoretically attenuate unfavorable cardiometabolic effects of hyperprolactinemia, some studies showed that even after menopause, prolactin excess is associated with increased cardiometabolic risk [11,12,13]. After menopause, prolactin concentration correlated with central and peripheral blood pressure, arterial stiffness, and with HeartScore, a composite index predicting 10-year cardiovascular mortality [11,12]. Higher daytime plasma prolactin concentration in postmenopausal women was an independent predictor of new-onset hypertension during 8 years of observation [13]. Moreover, some studies, including mainly middle-aged or elderly subjects, documented a relationship between prolactin concentrations and cardiovascular events [14,15,16]. Individuals with acute coronary syndromes were characterized by higher levels of this hormone in comparison to patients with stable angina pectoris. The highest concentrations were reported in the case of patients with acute myocardial infarction [14]. Moreover, higher prolactin concentrations were found to increase the risk of development of ischemic stroke and venous thromboembolism, in part by stimulating platelet activation [15,16].

A burgeoning body of research suggests that autoimmune thyroiditis (AT), often referred to as Hashimoto’s disease (HD), may predispose individuals to cardiometabolic problems, even in those with normal hypothalamic-pituitary-thyroid axis function. In women with a body mass index (BMI) of 25 kg/m^2^ or greater, hypertension was correlated with elevated carotid artery intima-media thickness, independent of thyroid function, BMI, and cardiovascular risk factors [17]. Euthyroid women with HD had diminished flow-mediated arterial dilation, although these abnormalities were less severe than those observed in hypothyroid women with AT [18]. HD was associated with increased pulse wave velocity in women, though this effect was less pronounced in postmenopausal than in premenopausal ones, possibly due to a more pronounced atherogenic profile [19]. Individuals with AT were found to have a lower mean reactive hyperemia index than healthy controls, and this difference was more pronounced in the case of positivity for thyroid peroxidase antibodies (TPOAbs) or for both TPOAbs and thyroglobulin antibodies (TgAbs) than for only TgAbs [20]. Thyroid autoimmunity was found to be positively associated with obesity, central obesity, glycated hemoglobin (HbA1c), the homeostatic model assessment 1 of insulin resistance ratio (HOMA1-IR), hyperlipidemia, and metabolic syndrome [21,22]. Moreover, women with euthyroid HD, the majority of whom were of reproductive age, were characterized by a more pronounced systemic proinflammatory state and more pronounced release of proinflammatory cytokines by stimulated human monocytes and lymphocytes [23]. Lastly, AT attenuated the impact of atorvastatin on circulating levels of cardiometabolic risk factors in women with hypercholesterolemia [24]. All these findings may partially explain why patients older than 50 years of age at diagnosis of AT showed nearly a threefold increase in cardiovascular hospitalizations [25]. The findings indicating elevated cardiometabolic risk in individuals with AT appear clinically significant, as AT is among the most prevalent human disorders, the foremost organ-specific autoimmune disorder, and a primary cause of hypothyroidism, which inherently heightens cardiometabolic risk [26,27,28,29].

Considering the high prevalence of AT and the relatively high prevalence of hyperprolactinemia, both disorders may coexist with each other, and at least theoretically, such patients may be at higher cardiometabolic risk in comparison to individuals with the presence of only one disorder. Recently, our research group has reported that cabergoline treatment of women at a reproductive age was associated with a less beneficial effect on cardiometabolic risk factors if they had concurrent thyroid autoimmunity [30]. However, coronary artery disease and stroke predominantly affect older women, while young women are generally characterized by a low risk of cardiovascular disease [31,32]. Moreover, the risk of cardiovascular admission was found to be increased in patients with AT older than 40 years old [25]. These findings suggest that cardiometabolic consequences of interactions between AT and cabergoline may be age-related and may differ between premenopausal and postmenopausal women. Consequently, the goal of the present investigation was to determine whether the cardiometabolic effects of cabergoline in women after menopause are influenced by coexistent thyroid autoimmunity.

## 2. Materials and Methods

The study protocol was endorsed by the institutional committee on human research, and all procedures were conducted in accordance with the Declaration of Helsinki. All participants provided written informed consent prior to inclusion. Registration at a clinical trial registry was not necessary due to the study design, which did not involve randomization and the same treatment.

### 2.1. Study Population

The subjects of this prospective cohort study were selected from postmenopausal women aged 50 to 75 years, exhibiting increased prolactin levels (exceeding 30 ng/mL). Women satisfied the inclusion criteria if more than 12 months had elapsed since their last menstruation, and hormone levels were within the menopausal range (FSH concentration surpassed 30 IU/L and estradiol concentration was below 30 pg/mL) on at least two occasions, separated by a minimum of four weeks. Standard cut-off values were employed to delineate both diabetes and prediabetes [30]. Enrollment was restricted to people who adhered to lifestyle advice for a minimum of 12 weeks. Participants were categorized into one of two groups according to their thyroid status. Patients assigned to group 1 were required to meet the following criteria for euthyroid autoimmune thyroiditis: (a) TPOAbs exceeding 100 U/mL; (b) the characteristic ultrasound morphology of the thyroid gland; (c) plasma levels of thyroid-stimulating hormone (TSH) and free thyroid hormones within the normal reference range (TSH between 0.4 and 4.5 mU/L, free thyroxine between 10.0 and 22.0 pmol/L, and free triiodothyronine between 2.3 and 6.5 pmol/L). Group 2 comprised patients devoid of thyroid disease, characterized by normal antibody titers, TSH levels, thyroid hormone levels, and a standard ultrasonography pattern of the thyroid gland. A priori sample size calculation revealed that the estimated sample size required for a given power (80%), confidence (95%), and effect size (20% difference in prolactin levels) was 22 individuals per group. Considering possible withdrawals and/or losses during the study, we included a greater number of patients (25 per group). In order to match the study groups for age, body mass index (BMI) and prolactin concentration, the number of recruited patients was lower than the total number of women meeting the study criteria. This procedure was based on the minimum Euclidean distance calculation. In order to minimize the impact of seasonal variations in the assessed variables, nearly half of patients (12 in each group) began cabergoline treatment between mid-October and mid-December, while the remaining ones (*n* = 26) between mid-April and mid-June.

Patients taking any drugs (with the exception of drugs causing hyperprolactinemia) were not considered for enrollment. We also excluded subjects with macroprolactinemia, positive antibodies against thyrotropin receptor, diabetes requiring pharmacotherapy, other autoimmune or endocrine disorders, cardiovascular disorders (except for grade 1 hypertension), hepatic or renal insufficiency, malabsorption syndromes, or other serious disorders.

### 2.2. Study Design

**Figure 1:**
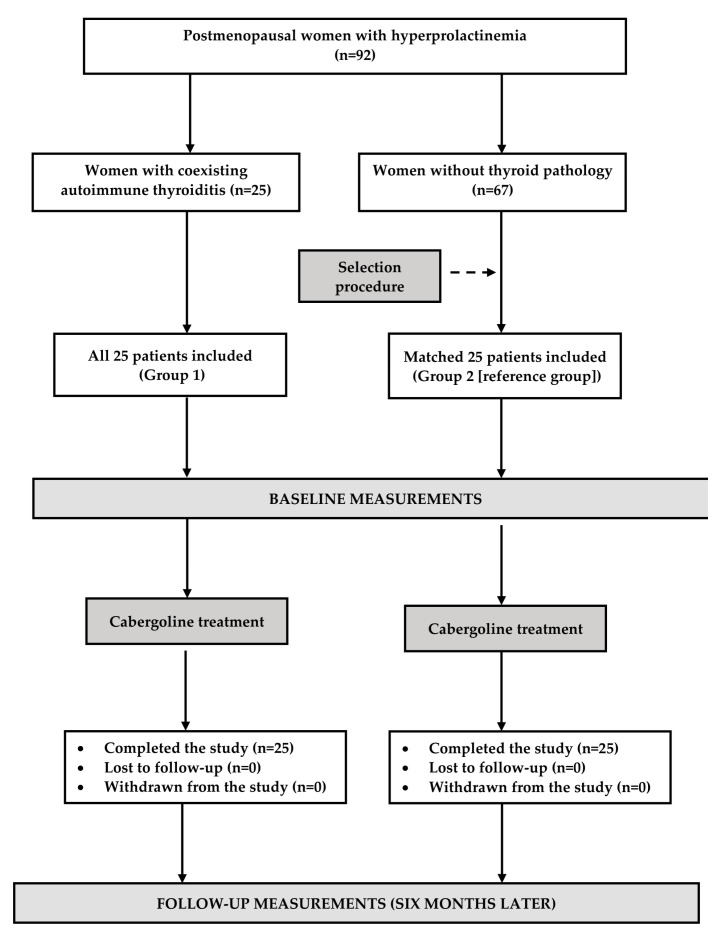
Flow the patients through the study.

The flow of patients through the study is depicted in Figure 1. Throughout the study, all patients received oral cabergoline. The starting dose for this agent was 0.25 mg once weekly, during or immediately after the evening meal. After two weeks, daily dosing was increased to a target dose of 0.25 mg twice weekly, taken until the end of the study. In patients with iatrogenic hyperprolactinemia, there were no changes in dosage of drugs responsible for prolactin excess. Over the entire study period, all patients kept on non-pharmacological management, started before the study began. The use of other drugs was allowed only if the treatment lasted for less than a week and was terminated no later than a month before the final (follow-up) visit. Compliance was measured once in two months by counting unused tablets and analysis of eating diaries and self-reports of physical activity. The 10-year cardiovascular risk was calculated using the Framingham Risk Score (FRS).

### 2.3. Laboratory Assays

All laboratory assays were conducted at baseline and six months subsequently. Venous blood samples were collected from the antecubital vein in the morning while seated for at least 30 min following an overnight fast. The experiments were conducted in duplicate to verify reproducibility. The plasma concentrations of glucose, lipids (total cholesterol, low-density lipoprotein [LDL] cholesterol, high-density lipoprotein [HDL] cholesterol, and triglycerides), and uric acid, as well as the urinary concentrations of albumin and creatinine, were assessed using commercial kits and standardized methods (Roche Diagnostics, Basel, Switzerland). The urine albumin-to-creatinine ratio (UACR) is determined by dividing the concentration of urinary albumin by the concentration of urinary creatinine. HbA1c was quantified in whole blood specimens utilizing the multi-analyzer (COBAS Integra, Roche Diagnostics, Mannheim, Germany). Plasma titers of TPOAbs and TgAbs, along with quantities of prolactin, insulin, TSH, and homocysteine, were quantified utilizing acridinium ester technology (ADVIA Centaur XP Immunoassay System, Siemens Healthcare Diagnostics, Munich, Germany). The concentration of prolactin was assessed prior to (total prolactin) and subsequent to polyethylene glycol precipitation [31,33,34]. The homeostatic model assessment 1 of insulin resistance (HOMA1-IR) is determined by multiplying glucose (mg/dL) by insulin (mU/L) and dividing the resultant product by 405. Plasma concentrations of high-sensitivity C-reactive protein (hsCRP) were evaluated using an immunoassay with chemiluminescent detection (Immulite 2000XPi, Siemens Healthcare, Warsaw, Poland). Fibrinogen was measured using the Clauss method with the BCS XP coagulometer (Siemens Healthcare, Warsaw, Poland).

### 2.4. Statistical Analysis

Prior to statistical analysis, all data were subjected to log-transformation to achieve normality and homogeneity. Differences between the groups were compared across each variable using Student’s *t*-tests for independent samples. Data at baseline and follow-up were compared using Student’s paired *t*-test. The χ^2^ test was used to assess differences in the distribution of categorical variables. The relationships between the outcome variables were analyzed using Pearson’s correlation coefficient. To identify factors that are important for increased cardiometabolic risk, logistic regression analysis was performed with elevated (above the reference range) levels of more than half of the investigated biomarkers as the dependent variable, and the presence of AT, systemic inflammation, prolactin excess and the conditions resulting in hyperprolactinemia as the independent variables. Unless otherwise stated, *p*-values below 0.05 (after correlation for multiple testing) indicated statistical significance.

## 3. Results

At study entry, there were no differences between both groups of patients with hyperprolactinemia in terms of age, smoking, reasons for hyperprolactinemia, the percentage of patients with prediabetes and type 2 diabetes (non-pharmacologically treated), BMI and blood pressure (both systolic and diastolic) (Table 1). There were between-group differences in HOMA1-IR, HDL cholesterol, antibody titers, uric acid, hsCRP, fibrinogen, homocysteine, and UACR. The study groups did not differ in prolactin (total and monomeric), fasting glucose, HbA1c, total cholesterol, LDL cholesterol, triglycerides, FRS and TSH (Table 2). Logistic regression analysis showed that AT (odds ratio [OR]: 3.20, 95% confidence interval [CI]: 1.10–5.53), hyperprolactinemia (OR: 3.41, 95% CI: 1.22–5.82) and elevated levels of hsCRP (OR: 1.91, 95% CI: 1.07–4.02) were significant determinants of increased cardiometabolic risk.

Adverse effects of cabergoline treatment were reported in four participants (two patients in each group) (8%). who complained of nausea, constipation, tiredness, and general weakness. However, these side effects were mild in intensity and transient. The remaining 46 patients (92%) did not report any adverse effects. Because no patient was withdrawn, the outcome variables of all included subjects were subjected to statistical analysis. Over the entire study period, all participants adhered to pharmacological and non-pharmacological treatment.

Cabergoline treatment did not affect BMI, systolic blood pressure, and diastolic blood pressure, as well as there were no differences in follow-up values of these parameters between both study groups (Table 1).

In both study groups, cabergoline decreased total and monomeric prolactin, HOMA1-IR, and UACR, and increased HDL cholesterol. In group 2, but not in group 1, the drug reduced triglycerides, uric acid, hsCRP, fibrinogen, homocysteine, and FRS. Cabergoline did not affect fasting glucose, HbA1c, antibody titers, and TSH. At the end of the study, both groups differed in prolactin (both total and monomeric), HOMA1-IR, thyroid antibody titers, uric acid, hsCRP, fibrinogen, homocysteine, UACR, and FRS (Table 2).

**Figure 2:**
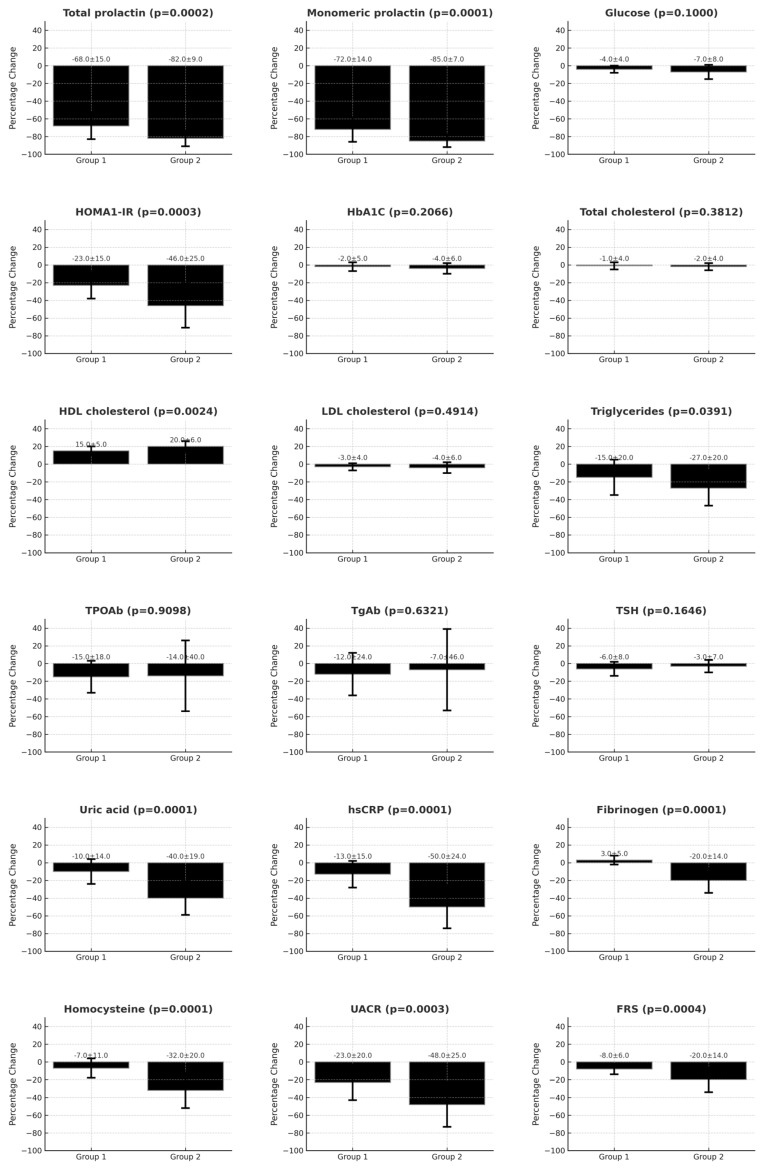
Percentage changes from baseline in the investigated variables during cabergoline treatment. Group 1: women with prolactin excess and AT. Group 2: women with prolactin excess but without thyroid pathology. The Supplementary Files (Table S1) present the full data as the mean and standard deviation. Abbreviations: AT—autoimmune thyroiditis; FRS—Framingham Risk Score; HbA1c—glycated hemoglobin; HDL—high-density lipoprotein; HOMA1-IR—the homeostatic model assessment 1 of insulin resistance ratio; hsCRP—high-sensitivity C-reactive protein; LDL—low-density lipoprotein; TgAbs—thyroglobulin antibodies; TPOAbs—thyroid peroxidase antibodies; TSH—thyroid-stimulating hormone; UACR—urinary albumin-to-creatinine ratio.

There were between-group differences in the percent changes from baseline in total prolactin, monomeric prolactin, HOMA1-IR, HDL cholesterol, triglycerides, uric acid, hsCRP, fibrinogen, homocysteine, UACR, and FRS, which were more pronounced in group 2 than group 1 (Figure 2).

At study entry, prolactin concentrations positively correlated with HOMA1-IR, uric acid, hsCRP, fibrinogen, homocysteine, UACR, and FRS (r values between 0.295 [*p* = 0.0455] and 0.406 [*p* = 0.0008] for total prolactin and between 0.307 [*p* = 0.0417] and 0.422 [*p* = 0.0006] for monomeric prolactin). If only group 1 was analyzed, hsCRP levels positively correlated with thyroid antibodies (TPOAbs: r = 0.501, *p* = 0.0001; TgAbs: r = 0.421, *p* = 0.0006), HOMA1-IR (r = 0.345, *p* = 0.0246), uric acid (r = 0.341, *p* = 0.0265), fibrinogen (r = 0.322, *p* = 0.0348), homocysteine (r = 0.381, *p* = 0.0019), UACR (r = 0.400, *p* < 0.0008), and FRS (r = 0.328 *p* = 0.0295). The impact of cabergoline on HOMA1-IR, HDL cholesterol, triglycerides, uric acid, hsCRP, fibrinogen, homocysteine, UACR, and FRS positively correlated with the reduction in total prolactin (group 1—r values between 0.314 [*p* = 0.0364] and 0.392 [*p* = 0.0012], group 2—r values between 0.318 [*p* = 0.0325] and 0.432 [*p* = 0.0008]) and in monomeric prolactin (group 1—r values between 0.302 [*p* = 0.0395] and 0.386 [*p* = 0.0024], group 2—r values between 0.341 [*p* = 0.0221] and 0.439 [*p* = 0.0006]). In both groups, the strongest correlations were reported for changes in hsCRP. In group 1, the impact of treatment on hsCRP inversely correlated with titers of TPOAbs (r = −0.422, *p* < 0.0006) and TgAbs (r = −0.401, *p* < 0.0010). Moreover, in this group of patients, the impact on HOMA1-IR, HDL cholesterol, triglycerides, uric acid, fibrinogen, homocysteine, UACR and FRS inversely correlated with baseline hsCRP concentration (r values between −0.265 [*p* = 0.0488] and −0.374 [*p* = 0.0056]) and positively correlated with the impact of cabergoline on hsCRP (r values between 0.255 [*p* = 0.0495] and 0.411 [*p* = 0.0011]). Other correlations did not reach statistical significance.

## 4. Discussion

Cabergoline administered to hyperprolactinemic women without thyroid pathology improved insulin sensitivity and plasma lipids, and reduced uric acid, hsCRP, fibrinogen, homocysteine, UACR, and FRS. These findings are in line with previous observations suggesting the cardiometabolic benefits of this drug in subjects with elevated prolactin concentrations [2,3,4,5,6,7]. Interestingly, these effects correlated with the degree of reduction in total and monomeric prolactin levels, suggesting that the cardiometabolic effects of cabergoline are secondary to its inhibitory effect on overactive lactotrophs. Considering correlations between prolactin levels and concentrations of risk factors at baseline, cardiometabolic benefits of cabergoline therapy seem to be important from the clinical point of view, suggesting that postmenopausal women may benefit from cabergoline treatment, irrespectively of the presence of symptoms of prolactin excess. Thus, it seems that dopamine agonists, both ergot-derived (cabergoline and bromocriptine) or non-ergot-derived (quinagolide and ropinirole), should be routinely prescribed to middle-aged or elderly women with hyperprolactinemia, irrespectively of its origin, even if they are asymptomatic or oligosymptomatic.

Another interesting finding is baseline differences between both study populations in most outcome measures: HOMA1-IR, HDL cholesterol, antibody titers, uric acid, hsCRP, fibrinogen, homocysteine, and UACR, suggesting that cardiometabolic risk may be greater if prolactin excess is observed in individuals with AT. Similar relationships were reported previously in studies including women in reproductive age [30,35], suggesting that additive effects of prolactin overproduction and AT on cardiometabolic risk in adult women are not determined by the impact of sex hormones. It should be underlined that between-group differences were observed despite matching both groups for BMI and prolactin levels. Theoretically, the differences would be greater in case of non-performance of this procedure, and in patients with more severe forms of HD, who were not included; however, to eliminate the potential effect of hypothyroidism, which is often a consequence of severe autoimmune thyroid disease. Moreover, strict inclusion and exclusion criteria do not allow us to explain our findings by the impact of comorbidities or co-medications. This finding may have practical implications. There are arguments indirectly suggesting that a high coincidence of prolactin excess and HD cannot be explained exclusively by their high prevalence in the general population. Firstly, via specific receptors, expressed on the surface of all immune cells, prolactin stimulates the maturation of T cells, impairs the negative selection of autoreactive B lymphocytes (promoting their proliferation, survival, and antibody production), and increases secretion of proinflammatory cytokines [36,37]. Secondly, enhanced prolactin production may be implicated in the development and perpetuation of many autoimmune disorders, and AT seems to be one of them [37]. Thirdly, thyroid antibodies were more prevalent in individuals with untreated hyperprolactinemia than in hyperprolactinemic subjects receiving specific treatment [38]. Similar relationships were observed if comparisons were limited to untreated and cabergoline-treated patients with prolactin-secreting tumors [39]. Lastly, mild or moderate prolactin excess is diagnosed even in two-fifths of subjects with overt and in more than one-fifth of subjects with subclinical thyroid hypofunction [40], which in developed countries is most often secondary to HD [26,27,28]. Thus, it seems that the optimal treatment of patients with both these disorders should be directed at reducing both prolactin oversecretion and thyroid autoimmunity.

Unfortunately, cabergoline treatment of hyperprolactinemic women with AT, though well tolerated, was associated with less expressed benefits than the same treatment of matched hyperprolactinemic women without thyroid pathology, despite the same cabergoline dose (0.5 mg weekly) and treatment duration (six months) in both groups. Moreover, the drug produced a neutral effect on thyroid antibody titers in the subgroup with AT, which is in disagreement with previous observations [38,39]. However, unlike earlier studies, our study was prospective, and limited only to women; the study population was more homogenous, and did not include subjects on other therapies (except for drugs increasing prolactin concentrations). Considering moderate positive correlations between the degree of prolactin reduction and the changes in cardiometabolic risk factors and between-group differences in follow-up concentrations of total and monomeric prolactin, it seems that the differences in cardiometabolic effects were in part secondary to a weaker effect of the studied drug on lactotroph secretory function. Theoretically, higher doses of cabergoline would be more efficient, and we intend to verify this interesting hypothesis in our future studies.

Weaker cardiometabolic effects of cabergoline in women with HD may be, at least in part, explained by low-grade systemic inflammation associated with AT. Apart from impaired endothelial function, increased adipose tissue content, insulin resistance, and increased procoagulant activity, chronic inflammation is also postulated to be one of the most important mechanisms leading to cardiovascular alterations in hyperprolactinemia [2,3,4,5,6,7,8,9]. The treatment-induced decrease in hsCRP levels in women with AT positively linked with cabergoline’s effect on other cardiometabolic risk variables, whereas the extent of reduction was inversely correlated with initial hsCRP levels. These relationships align with prior studies indicating that proinflammatory mediators enhance the synthesis of uric acid, fibrinogen, homocysteine, and UACR [37,38,39,40,41,42,43,44]. Notably, women with AT exhibited positive correlations between baseline hsCRP levels and baseline antibody titers, as well as inverse correlations between the reduction in hsCRP and titers of TPOAbs and TgAbs; however, no correlations were found between the decrease in hsCRP and TSH, which remained within normal limits for all participants. The results indicate that the mitigating influence of AT on the cardiometabolic consequences of cabergoline is contingent upon the degree of thyroid autoimmune disease, although appears it independent of the activity of the hypothalamic-pituitary-thyroid axis. Consequently, the cardiometabolic effects of cabergoline in women are expected to be more significant in less severe cases of AT. Furthermore, these effects may be more evident in individuals concurrently administered agents that diminish thyroid antibody titers, such as vitamin D, selenomethionine, and myo-inositol, or in conjunction with other therapies targeting C-reactive protein, including cyclooxygenase inhibitors, statins, angiotensin-converting enzyme inhibitors, certain sartans, or antioxidants [45,46].

The obtained results are in line with previous observations of our research team, indicating that AT mitigated the inhibitory effect of metformin on prolactin and gonadotropin secretion by overactive pituitary cells in patients with prediabetes [35,47]. This is another argument in favor of interaction between thyroiditis and cabergoline, also at the level of the pituitary lactotrophs or the upstream dopaminergic neurons. This explanation is in line with the finding that experimental inflammation was found to increase pituitary prolactin production by an inhibitory effect on the activity of the tuberoinfundibular dopaminergic system [48]. Thus, inflammation-induced hypofunction of this pathway may attenuate the impact of dopamine agonists, including cabergoline.

There are also other conclusions that can be drawn. Firstly, considering that even in apparently healthy adult populations cardiometabolic risk increases with age [49], this risk in postmenopausal hyperprolactinemic women with AT is probably greater than if both disorders affect women of reproductive age, and obligatory treatment of such patients should be considered. Secondly, assessment of prolactin concentration and hsCRP during the follow-up may provide an indirect insight into the cardiometabolic effects of cabergoline in females with coexistent prolactin excess and thyroid autoimmunity. Lastly, the unfavorable effect of prolactin excess on cardiometabolic risk can be explained by the impact of the monomeric form of this hormone but not by high-molecular-weight forms of prolactin (macroprolactin) [50]. Because of parallel changes in total and monomeric prolactin and similar correlations with cardiometabolic risk factors, it seems that small amounts of circulating complexes of prolactin and immunoglobulins and aggregates of polymers of this hormone, present in almost all subjects, do not seem to affect levels of cardiovascular risk factors.

Despite a neutral effect on BMI, fasting glucose, HbA1c, and blood pressure, cabergoline improved insulin sensitivity in both study groups, though this effect was stronger in women without thyroid pathology. This observation may be explained by the interaction between cabergoline and low-grade inflammation at the level of GLUT4, the main insulin-responsive glucose transporter [51]. In line with this explanation, the expression of GLUT4 was stimulated by dopaminergic drugs [52], while the opposite effect was induced by proinflammatory cytokines [53,54,55]. These differences in insulin sensitivity may also explain baseline differences in HDL cholesterol and a stronger effect of cabergoline on HDL cholesterol and triglycerides in individuals without thyroid pathology, contrasting with the neutral effect on total and LDL cholesterol [56].

Some study limitations deserve to be mentioned. The study design may have resulted in the obtained results being affected by selection and confounding bias. The limited sample size complicates the ability to make extensive inferences from the study. We solely assessed surrogate outcomes as proxies for clinical endpoints. The existence of numerous confounding variables may complicate the differentiation of causation from correlation. Precautions implemented during study design and data processing were limited, although they did not entirely eradicate the regression toward the mean phenomena [57]. The study’s enrollment of patients with hyperprolactinemia from various origins precludes the complete exclusion of the possibility that the influence of AT on the cardiometabolic effects of cabergoline may vary according to the underlying cause of prolactin excess.

## 5. Conclusions

The impact of cabergoline on cardiometabolic risk factors in hyperprolactinemic women after menopause is less pronounced in the case of coexistent thyroid autoimmunity. The difference in the strength of action seems to be explained in part by a stronger effect on prolactin concentration in women without thyroid pathology than in women with AT. The unfavorable effect of thyroid autoimmunity on cabergoline action is probably also associated with the impact of inflammation at the level of the anterior pituitary, but not with the changes in activity of the hypothalamus-pituitary-thyroid axis. Given the potential practical significance of our findings but also methodological limitations, the obtained results require confirmation in subsequent studies, including a larger population of patients. Further research should also identify the remaining comorbidities influencing the pharmacodynamic effects of cabergoline and the remaining dopamine agonists.

## Figures and Tables

**Table 1:** Baseline and follow-up characteristics of both study groups.

Variable	Group 1	Group 2	*p*-Value
Number (n)	25	25	-
Age (years)	63 ± 6	62 ± 7	0.5585
Smokers (%)/number of cigarettes a day (n)/duration of smoking (years)	40/10 ± 6/34 ± 15	36/9 ± 6/32 ± 14	0.4215
Reasons for prolactin excess: drug-induced hyperprolactinemia/prolactinoma/empty sella syndrome/brain injury/idiopathic hyperprolactinemia (%)	36/16/24/12/12	40/12/20/16/12	0.7528
Prediabetes/type 2 diabetes * (%)	56/8	48/4	0.1401
BMI (kg/m^2^)			
Baseline	25.3 ± 4.6	24.9 ± 4.3	0.7522
Follow-up	25.0 ± 4.8	23.6 ± 4.2	0.2780
*p*-value (follow-up vs. baseline)	0.8225	0.2829	-
Systolic blood pressure (mmHg)			
Baseline	134 ± 15	131 ± 17	0.5144
Follow-up	130 ± 15	125 ± 14	0.2290
*p*-value (follow-up vs. baseline)	0.3505	0.1795	-
Diastolic blood pressure (mmHg)			
Baseline	81 ± 5	80 ± 5	0.4829
Follow-up	80 ± 6	79 ± 5	0.5251
*p*-value (follow-up vs. baseline)	0.5251	0.4829	-

Group 1: women with prolactin excess and AT. Group 2: women with prolactin excess but without thyroid pathology. Except for smoking and reasons for prolactin excess, the data are presented as the mean ± standard deviation. * non-pharmacologically treated. Abbreviations: AT—autoimmune thyroiditis; BMI—body mass index.

**Table 2:** The effect of cabergoline on the investigated variables in the study population.

Variable	Group 1	Group 2	*p*-Value (1 vs. 2)
Total prolactin (ng/mL)			
Baseline	68.2 ± 20.0	70.1 ± 24.2	0.7635
Follow-up	21.9 ± 8.8	12.9 ± 6.7	0.0002
*p*-value (follow-up vs. baseline)	<0.0001	<0.0001	-
Monomeric prolactin (ng/mL)			
Baseline	64.2 ± 10.8	67.1 ± 23.5	0.5776
Follow-up	18.1 ± 6.4	10.1 ± 5.9	<0.0001
*p*-value (follow-up vs. baseline)	<0.0001	<0.0001	-
Glucose (mg/dL)			
Baseline	102 ± 15	104 ± 14	0.6282
Follow-up	98 ± 13	97 ± 14	0.7950
*p*-value (follow-up vs. baseline)	0.3187	0.0835	-
HOMA1-IR			
Baseline	3.5 ± 1.4	2.8 ± 1.0	0.0475
Follow-up	2.7 ± 1.2	1.5 ± 0.8	0.0001
*p*-value (follow-up vs. baseline)	0.0350	<0.0001	-
HbA1c (%)			
Baseline	5.6 ± 0.5	5.5 ± 0.5	0.4829
Follow-up	5.5 ± 0.6	5.3 ± 0.5	0.2066
*p*-value (follow-up vs. baseline)	0.5251	0.1638	-
Total cholesterol (mg/dL)			
Baseline	192 ± 48	198 ± 65	0.7121
Follow-up	190 ± 55	195 ± 62	0.7642
*p*-value (follow-up vs. baseline)	0.8916	0.8681	-
HDL-cholesterol (mg/dL)			
Baseline	41 ± 8	46 ± 8	0.0319
Follow-up	47 ± 9	55 ± 9	0.0029
*p*-value (follow-up vs. baseline)	0.0162	0.0005	-
LDL-cholesterol (mg/dL)			
Baseline	115 ± 34	118 ± 24	0.7202
Follow-up	112 ± 31	113 ± 35	0.9153
*p*-value (follow-up vs. baseline)	0.7458	0.5586	-
Triglycerides (mg/dL)			
Baseline	164 ± 53	171 ± 70	0.6918
Follow-up	140 ± 67	125 ± 52	0.3809
*p*-value (follow-up vs. baseline)	0.0668	0.0112	-
TPOAb (U/mL)			
Baseline	832 ± 345	14 ± 8	<0.0001
Follow-up	705 ± 267	12 ± 8	<0.0001
*p*-value (follow-up vs. baseline)	0.1411	0.3812	-
TgAb (U/mL)			
Baseline	785 ± 371	16 ± 10	<0.0001
Follow-up	690 ± 288	15 ± 12	<0.0001
*p*-value (follow-up vs. baseline)	0.3169	0.7503	-
TSH (mU/L)			
Baseline	3.1 ± 0.7	2.9 ± 0.9	0.3848
Follow-up	2.9 ± 0.8	2.8 ± 1.0	0.6970
*p*-value (follow-up vs. baseline)	0.3516	0.7118	-
Uric acid (mg/dL)			
Baseline	4.0 ± 1.4	4.8 ± 1.2	0.0350
Follow-up	3.6 ± 1.3	2.9 ± 0.7	0.0218
*p*-value (follow-up vs. baseline)	0.3004	<0.0001	-
hsCRP (mg/L)			
Baseline	3.9 ± 1.2	3.0 ± 1.3	0.0142
Follow-up	3.4 ± 1.3	1.5 ± 0.8	<0.0001
*p*-value (follow-up vs. baseline)	0.1641	<0.0001	-
Fibrinogen (mg/dL)			
Baseline	402 ± 80	350 ± 101	0.0492
Follow-up	416 ± 121	281 ± 88	<0.0001
*p*-value (follow-up vs. baseline)	0.6316	0.0131	-
Homocysteine (μmol/L)			
Baseline	30.9 ± 11.0	24.7 ± 9.5	0.0381
Follow-up	28.6 ± 12.2	16.8 ± 8.0	0.0002
*p*-value (follow-up vs. baseline)	0.4873	0.0026	-
UACR (mg/g)			
Baseline	29.5 ± 10.3	23.0 ± 9.2	0.0228
Follow-up	22.8 ± 8.5	11.9 ± 5.6	<0.0001
*p*-value (follow-up vs. baseline)	0.0156	<0.0001	-
FRS (%)			
Baseline	9.6 ± 2.3	9.1 ± 2.2	0.4360
Follow-up	8.8 ± 2.0	7.3 ± 2.0	0.0108
*p*-value (follow-up vs. baseline)	0.1956	0.0040	-

Group 1: women with prolactin excess and AT. Group 2: women with prolactin excess but without thyroid pathology. The data are presented as the mean ± standard deviation. Abbreviations: AT—autoimmune thyroiditis; FRS—Framingham Risk Score; HbA1c—glycated hemoglobin; HDL—high-density lipoprotein; HOMA1-IR—the homeostatic model assessment 1 of insulin resistance ratio; hsCRP—high-sensitivity C-reactive protein; LDL—low-density lipoprotein; TgAbs—thyroglobulin antibodies; TPOAbs—thyroid peroxidase antibodies; TSH—thyroid-stimulating hormone; UACR—urinary albumin-to-creatinine ratio.

## Data Availability

The data that support the findings of this study are available from the corresponding author upon reasonable request.

## Supplementary Materials

The following supporting information can be downloaded at: https://www.mdpi.com/article/10.3390/metabo15010009/s1, Table S1: Percentage changes from baseline in the investigated variables during cabergoline treatment.
