# Supplementary material for: The Influence of Concurrent Autoimmune Thyroiditis on the Cardiometabolic Consequences of Cabergoline in Postmenopausal Women
Source: Metabolites. 2025 Jan 1;15(1):9. doi: 10.3390/metabo15010009 (PMC11767027; doi:10.3390/metabo15010009)
Supplement: Supplementary file 1 [file metabolites-15-00009-s001.zip › metabolites-3358490-supplementary.pdf]

Table S1. Percentage changes from baseline in the investigated variables during cabergoline treatment

Variable	Group 1	Group 2	*p*-value
Δ Total prolactin	-68±15	-82±9	0.0002
Δ Monomeric prolactin	-72±14	-85±7	0.0001
Δ Glucose	-4±4	-7±8	0.1000
Δ HOMA1-IR	-23±15	-46±25	0.0003
Δ HbA_{1C}	-2±5	-4±6	0.2066
Δ Total cholesterol	-1±4	-2±4	0.3812
Δ HDL cholesterol	15±5	20±6	0.0024
Δ LDL cholesterol	-3±4	-4±6	0.4914
Δ Triglycerides	-15±20	-27±20	0.0391
Δ TPOAb	-15±18	-14±40	0.9098
Δ TgAb	-12±24	-7±46	0.6321
Δ TSH	-6±8	-3±7	0.1646
Δ uric acid	-10±14	-40±19	<0.0001
Δ hsCRP	-13±15	-50±24	<0.0001
Δ Fibrinogen	3±5	-20±14	<0.0001
Δ Homocysteine	-7±11	-32±20	<0.0001
Δ UACR	-23±20	-48±25	0.0003
Δ FRS	-8±6	-20±14	0.0004

Group 1: women with prolactin excess and AT. Group 2: women with prolactin excess but without thyroid pathology. The data are presented as the mean ± standard deviation. *Abbreviations: AT – autoimmune thyroiditis; FRS - Framingham Risk Score; HbA_{1C} - glycated hemoglobin; HDL - high-density lipoprotein; HOMA1-IR - the homeostatic model assessment 1 of insulin resistance ratio; hsCRP - high-sensitivity C-reactive protein; LDL - low-density lipoprotein; TgAb - thyroglobulin antibodies; TPOAb - thyroid peroxidase antibodies; TSH - thyroid-stimulating hormone; UACR - urinary albumin-to-creatinine ratio*
